# Development of air conditioning technologies to reduce CO_2 _emissions in the commercial sector

**DOI:** 10.1186/1750-0680-1-12

**Published:** 2006-10-25

**Authors:** Yukiko Yoshida

**Affiliations:** 1 Center for Global Environmental Research, National Institute for Environmental Studies, 16-2 Onogawa, Tsukuba, Japan

## Abstract

**Background:**

Architectural methods that take into account global environmental conservation generally concentrate on mitigating the heat load of buildings. Here, we evaluate the reduction of carbon dioxide (CO_2_) emissions that can be achieved by improving heating, ventilating, and air conditioning (HVAC) technologies.

**Results:**

The Climate Change Research Hall (CCRH) of the National Institute for Environmental Studies (NIES) is used as a case study. CCRH was built in line with the "Green Government Buildings" program of the Government Buildings Department at the Ministry of Land, Infrastructure and Transport in Japan. We have assessed the technology used in this building, and found that there is a possibility to reduce energy consumption in the HVAC system by 30%.

**Conclusion:**

Saving energy reduces CO_2 _emissions in the commercial sector, although emission factors depend on the country or region. Consequently, energy savings potential may serve as a criterion in selecting HVAC technologies with respect to emission reduction targets.

## Background

Heating, ventilating, air conditioning (HVAC) and lighting systems in buildings are a major source of carbon dioxide (CO_2_) emissions in the commercial sector. Reduction of this source is a common issue for Asian countries that share similar constraints in developing solutions [1,2].

In Japan, a cooperative academic, industrial, and governmental project has been established to develop a new system called the Comprehensive Assessment System for Building Environmental Efficiency (CASBEE). It evaluates all forms of energy usage within buildings [3].

The Climate Change Research Hall (CCRH: completed in 2001, ferroconcrete, three floors, 4900 m^2 ^total floor space) at the National Institute for Environmental Studies in Japan was constructed according to the latest sustainable environment designs, including global warming abatement technology for buildings [4].

Consumption of electricity for lighting, which is related to the interior heat generation load, changes little from season to season. This building is equipped with 32 W high-frequency fluorescent lights whose intensity is controlled to keep a constant brightness independent of the outdoor brightness. The automatic control of lighting was found to realize approximately 30% in annual energy savings compared to lighting with no automatic control [5].

**Figure 1:**
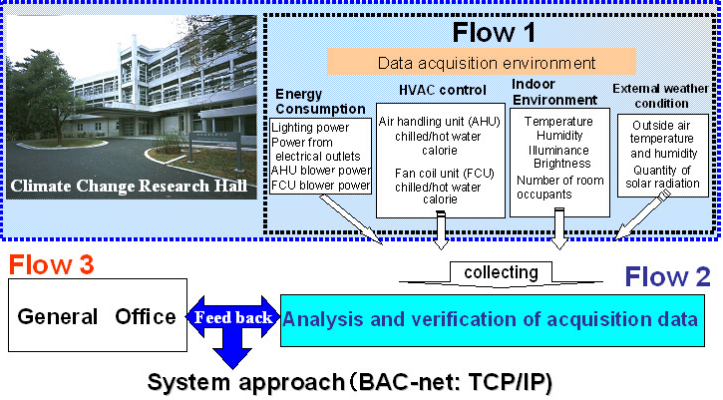
The system composition of CCRH.

Here, we assess the HVAC technologies used throughout this building (Figure 1) by quantifying the mitigation of the environmental burden achieved by using these technologies and management options as related to technology, institutions and culture [6].

## Results

**Figure 2:**
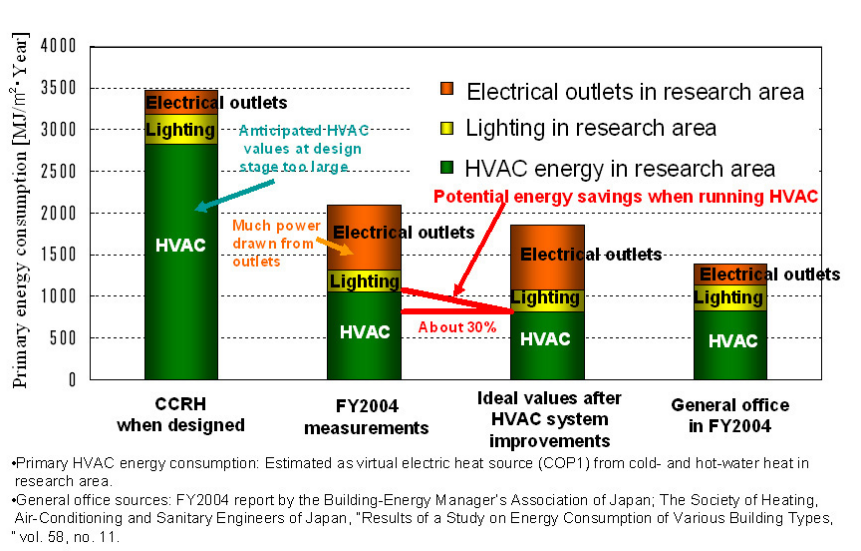
Primary energy consumption of CCRH.

Proceeding from data on energy consumption, HVAC control, and the indoor and outdoor environment, we developed a system for coordinating fan coil units (FCUs) and air-handing units (AHUs). Coordinated control and operation of FCUs and AHUs shuts down FCUs when the heat load through the windows is small. In rooms where occupants and other inside heat sources are fewer than anticipated by design, it imposes limits on excessive space cooling and uses AHU blower operation, which is more effective against room temperature increases than AHU cooling operation in the winter. This decreases energy loss and also provides comfort. According to calculations based on measurement data from the 2004 fiscal year, reducing the operating loss of FCUs and AHUs promises an HVAC energy reduction of about 30% (Figure 2).

**Figure 3:**
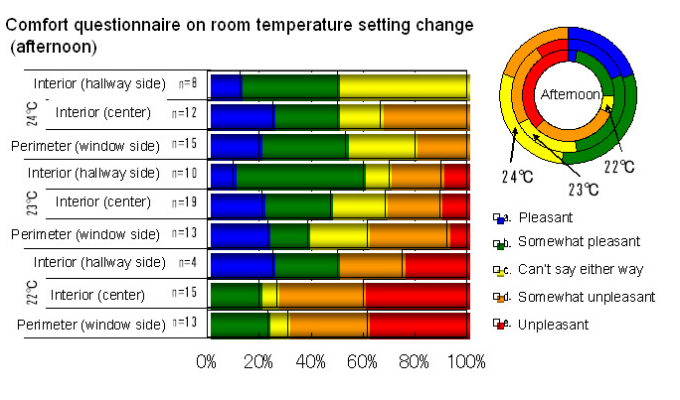
Winter comfort questionnaire.

In conjunction with changing the room temperature settings, we conducted a questionnaire on comfort, and obtained responses from over half of the room occupants. When the room temperature setting was lowered to 22°C, more respondents said they felt discomfort or slight discomfort than at 23°C. Also, regardless of the temperature to which the setting was changed, reports for the room perimeters were not as good as those for the interiors (Figure 3).

## Discussion

In the case of HVAC, the threshold between comfortable and uncomfortable conditions is very narrow. Control of temperature must be designed carefully in order to maximize energy reduction without imposing stress on the room occupants.

Accurate room temperature monitoring data should be fed back to the well-designed ventilation system based on a numerical model that takes into account the environmental and room use conditions. Such an intricate system designed specifically for an individual building will lead to maximum energy savings without disturbing the activities of its occupants.

Based on this concept, we evaluate energy savings potential (ESP) [7]:

ESP=Annual energy consumption from air-conditioningBaseline energy consumption from air-conditioning
 MathType@MTEF@5@5@+=feaafiart1ev1aaatCvAUfKttLearuWrP9MDH5MBPbIqV92AaeXatLxBI9gBaebbnrfifHhDYfgasaacH8akY=wiFfYdH8Gipec8Eeeu0xXdbba9frFj0=OqFfea0dXdd9vqai=hGuQ8kuc9pgc9s8qqaq=dirpe0xb9q8qiLsFr0=vr0=vr0dc8meaabaqaciaacaGaaeqabaqabeGadaaakeaacqqGfbqrcqqGtbWucqqGqbaucqGH9aqpfaqaaeGabqaabaGaeeyqaeKaeeOBa4MaeeOBa4MaeeyDauNaeeyyaeMaeeiBaWMaeeiiaaIaeeyzauMaeeOBa4MaeeyzauMaeeOCaiNaee4zaCMaeeyEaKNaeeiiaaIaee4yamMaee4Ba8MaeeOBa4Maee4CamNaeeyDauNaeeyBa0MaeeiCaaNaeeiDaqNaeeyAaKMaee4Ba8MaeeOBa4MaeeiiaaIaeeOzayMaeeOCaiNaee4Ba8MaeeyBa0MaeeiiaaIaeeyyaeMaeeyAaKMaeeOCaiNaeeyla0Iaee4yamMaee4Ba8MaeeOBa4MaeeizaqMaeeyAaKMaeeiDaqNaeeyAaKMaee4Ba8MaeeOBa4MaeeyAaKMaeeOBa4Maee4zaCgabaGaeeOqaiKaeeyyaeMaee4CamNaeeyzauMaeeiBaWMaeeyAaKMaeeOBa4MaeeyzauMaeeiiaaIaeeyzauMaeeOBa4MaeeyzauMaeeOCaiNaee4zaCMaeeyEaKNaeeiiaaIaee4yamMaee4Ba8MaeeOBa4Maee4CamNaeeyDauNaeeyBa0MaeeiCaaNaeeiDaqNaeeyAaKMaee4Ba8MaeeOBa4MaeeiiaaIaeeOzayMaeeOCaiNaee4Ba8MaeeyBa0MaeeiiaaIaeeyyaeMaeeyAaKMaeeOCaiNaeeyla0Iaee4yamMaee4Ba8MaeeOBa4MaeeizaqMaeeyAaKMaeeiDaqNaeeyAaKMaee4Ba8MaeeOBa4MaeeyAaKMaeeOBa4Maee4zaCgaaaaa@AE77@

in two ways,

ESP1=Annual energy consumption from air-conditioningBaseline energy consumption from air-conditioning according to greenhouse gas inventories
 MathType@MTEF@5@5@+=feaafiart1ev1aaatCvAUfKttLearuWrP9MDH5MBPbIqV92AaeXatLxBI9gBaebbnrfifHhDYfgasaacH8akY=wiFfYdH8Gipec8Eeeu0xXdbba9frFj0=OqFfea0dXdd9vqai=hGuQ8kuc9pgc9s8qqaq=dirpe0xb9q8qiLsFr0=vr0=vr0dc8meaabaqaciaacaGaaeqabaqabeGadaaakeaacqqGfbqrcqqGtbWucqqGqbaudaWgaaWcbaGaeeymaedabeaakiabg2da9uaabaqaceabaeaacqqGbbqqcqqGUbGBcqqGUbGBcqqG1bqDcqqGHbqycqqGSbaBcqqGGaaicqqGLbqzcqqGUbGBcqqGLbqzcqqGYbGCcqqGNbWzcqqG5bqEcqqGGaaicqqGJbWycqqGVbWBcqqGUbGBcqqGZbWCcqqG1bqDcqqGTbqBcqqGWbaCcqqG0baDcqqGPbqAcqqGVbWBcqqGUbGBcqqGGaaicqqGMbGzcqqGYbGCcqqGVbWBcqqGTbqBcqqGGaaicqqGHbqycqqGPbqAcqqGYbGCcqqGTaqlcqqGJbWycqqGVbWBcqqGUbGBcqqGKbazcqqGPbqAcqqG0baDcqqGPbqAcqqGVbWBcqqGUbGBcqqGPbqAcqqGUbGBcqqGNbWzaeaacqqGcbGqcqqGHbqycqqGZbWCcqqGLbqzcqqGSbaBcqqGPbqAcqqGUbGBcqqGLbqzcqqGGaaicqqGLbqzcqqGUbGBcqqGLbqzcqqGYbGCcqqGNbWzcqqG5bqEcqqGGaaicqqGJbWycqqGVbWBcqqGUbGBcqqGZbWCcqqG1bqDcqqGTbqBcqqGWbaCcqqG0baDcqqGPbqAcqqGVbWBcqqGUbGBcqqGGaaicqqGMbGzcqqGYbGCcqqGVbWBcqqGTbqBcqqGGaaicqqGHbqycqqGPbqAcqqGYbGCcqqGTaqlcqqGJbWycqqGVbWBcqqGUbGBcqqGKbazcqqGPbqAcqqG0baDcqqGPbqAcqqGVbWBcqqGUbGBcqqGPbqAcqqGUbGBcqqGNbWzcqqGGaaicqqGHbqycqqGJbWycqqGJbWycqqGVbWBcqqGYbGCcqqGKbazcqqGPbqAcqqGUbGBcqqGNbWzcqqGGaaicqqG0baDcqqGVbWBcqqGGaaicqqGNbWzcqqGYbGCcqqGLbqzcqqGLbqzcqqGUbGBcqqGObaAcqqGVbWBcqqG1bqDcqqGZbWCcqqGLbqzcqqGGaaicqqGNbWzcqqGHbqycqqGZbWCcqqGGaaicqqGPbqAcqqGUbGBcqqG2bGDcqqGLbqzcqqGUbGBcqqG0baDcqqGVbWBcqqGYbGCcqqGPbqAcqqGLbqzcqqGZbWCaaaaaa@E354@

ESP2=Annual energy consumption from air-conditioningBaseline energy consumption from air-conditioning under optimal HVAC control
 MathType@MTEF@5@5@+=feaafiart1ev1aaatCvAUfKttLearuWrP9MDH5MBPbIqV92AaeXatLxBI9gBaebbnrfifHhDYfgasaacH8akY=wiFfYdH8Gipec8Eeeu0xXdbba9frFj0=OqFfea0dXdd9vqai=hGuQ8kuc9pgc9s8qqaq=dirpe0xb9q8qiLsFr0=vr0=vr0dc8meaabaqaciaacaGaaeqabaqabeGadaaakeaacqqGfbqrcqqGtbWucqqGqbaudaWgaaWcbaGaeeOmaidabeaakiabg2da9uaabaqaceabaeaacqqGbbqqcqqGUbGBcqqGUbGBcqqG1bqDcqqGHbqycqqGSbaBcqqGGaaicqqGLbqzcqqGUbGBcqqGLbqzcqqGYbGCcqqGNbWzcqqG5bqEcqqGGaaicqqGJbWycqqGVbWBcqqGUbGBcqqGZbWCcqqG1bqDcqqGTbqBcqqGWbaCcqqG0baDcqqGPbqAcqqGVbWBcqqGUbGBcqqGGaaicqqGMbGzcqqGYbGCcqqGVbWBcqqGTbqBcqqGGaaicqqGHbqycqqGPbqAcqqGYbGCcqqGTaqlcqqGJbWycqqGVbWBcqqGUbGBcqqGKbazcqqGPbqAcqqG0baDcqqGPbqAcqqGVbWBcqqGUbGBcqqGPbqAcqqGUbGBcqqGNbWzaeaacqqGcbGqcqqGHbqycqqGZbWCcqqGLbqzcqqGSbaBcqqGPbqAcqqGUbGBcqqGLbqzcqqGGaaicqqGLbqzcqqGUbGBcqqGLbqzcqqGYbGCcqqGNbWzcqqG5bqEcqqGGaaicqqGJbWycqqGVbWBcqqGUbGBcqqGZbWCcqqG1bqDcqqGTbqBcqqGWbaCcqqG0baDcqqGPbqAcqqGVbWBcqqGUbGBcqqGGaaicqqGMbGzcqqGYbGCcqqGVbWBcqqGTbqBcqqGGaaicqqGHbqycqqGPbqAcqqGYbGCcqqGTaqlcqqGJbWycqqGVbWBcqqGUbGBcqqGKbazcqqGPbqAcqqG0baDcqqGPbqAcqqGVbWBcqqGUbGBcqqGPbqAcqqGUbGBcqqGNbWzcqqGGaaicqqG1bqDcqqGUbGBcqqGKbazcqqGLbqzcqqGYbGCcqqGGaaicqqGVbWBcqqGWbaCcqqG0baDcqqGPbqAcqqGTbqBcqqGHbqycqqGSbaBcqqGGaaicqqGibascqqGwbGvcqqGbbqqcqqGdbWqcqqGGaaicqqGJbWycqqGVbWBcqqGUbGBcqqG0baDcqqGYbGCcqqGVbWBcqqGSbaBaaaaaa@D143@

The total value of primary energy consumption in typical offices in the 2004 fiscal year was reported to be approximately 2000 MJ/m^2^/yr by the Building-Energy Manager's Association of Japan. This total amount approximates the values in the greenhouse gas inventory of Japan [8].

Annual HVAC energy consumption of a typical office in CCRH was 1058 MJ/m^2^/yr in 2004. However, according to the Building-Energy Manager's Association of Japan, general offices under HVAC control should have an annual consumption rate of 828 MJ/m^2^/yr. Therefore, we set the baseline energy consumption from air-conditioning to 828 MJ/m^2^/yr in accordance with the greenhouse gas inventories. Thus, ESP_1 _of CCRH was estimated to be 1.28 in 2004.

Baseline energy consumption under optimal HVAC control was estimated at 817 MJ/m^2^/yr in 2004 using an approach similar to that of Ishida and Mori [9]. This makes ESP_2 _equal to 1.30.

## Conclusion

Increasing awareness of environmental issues has led to development of a large number of energy conservation technologies for buildings, especially in more developed countries [4]. Energy savings potential (ESP) is a very important indicator for developing these technologies.

In Japan, attempts are being made to create policies such as the standards for planning eco-buildings by the Government Buildings Department at the Ministry of Land, Infrastructure and Transport. This document introduces approximately 50 methods and technologies and provides calculation formulas that can be evaluated using ESP.

Saving energy reduces CO_2 _emissions in the commercial sector, although emission factors depend on the country and region [1]. Therefore, we have to evaluate the methods developed in Japan for use in carbon management across the Asian region.

## Methods

**Figure 4:**
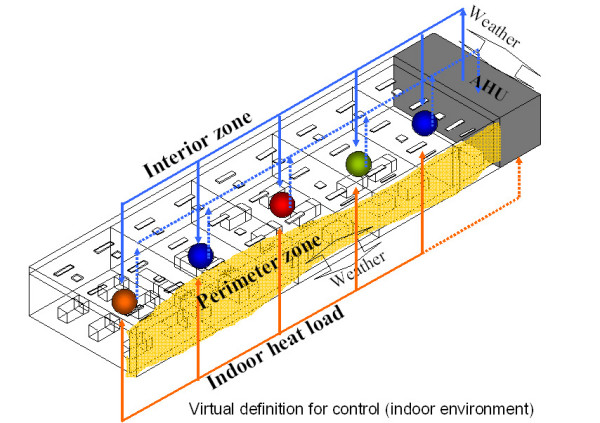
Virtual definition for control (indoor environment).

As in ordinary office buildings, the HVAC system of the building used in this research has FCUs on the window-side perimeter, while room interiors have AHU conditioning systems incorporating variable air volume (VAV) system control (Figure 4).

**Figure 5:**
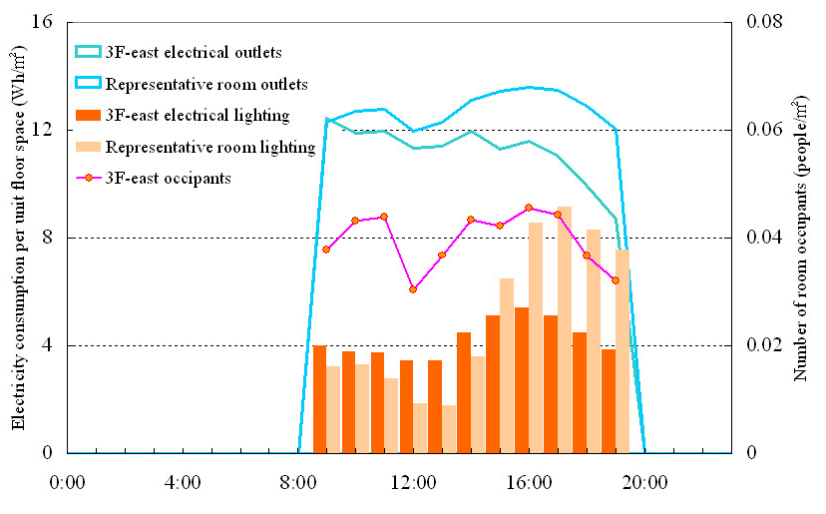
Electrical consumption from lighting, electrical outlets and number of room occupants.

The consumption of electricity for lighting, which is related to the interior heat generation load, changes little from season to season. The building in question is fitted with 32 W high-frequency fluorescent lighting with automatic lighting control performing initial intensity correction (Figure 5).

**Figure 6:**
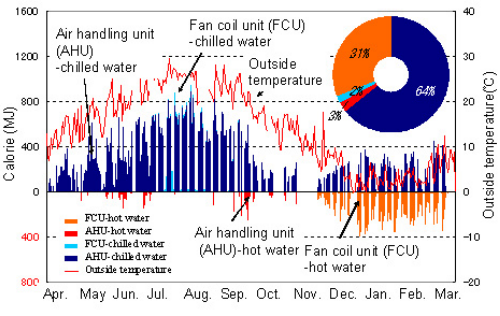
Annual energy consumption of HVAC system.

The energy consumption of the HVAC system is shown in Figure 6. On weekdays in the summer, cooling is achieved mainly with interior HVAC units (AHUs). When the outside temperature is 25°C or higher, window perimeter HVAC units (FCUs) are also used. In the winter, when the outside temperature is 10°C or lower, FCU heating and AHU cooling are used. There are concerns about energy operating loss due to simultaneous use of chilled and hot water, but it is hoped that energy will be saved through improvements to the system.

However, energy managers cannot determine whether a particular indoor environment is comfortable or not from the HVAC monitor, especially when users open the windows to let in outside air.

**Figure 7:**
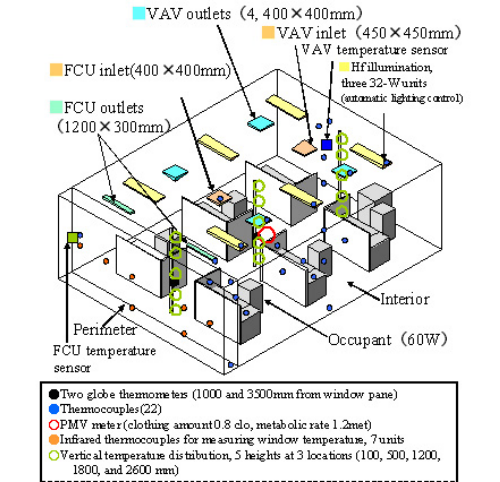
Detailed measurements in tested room.

**Figure 8:**
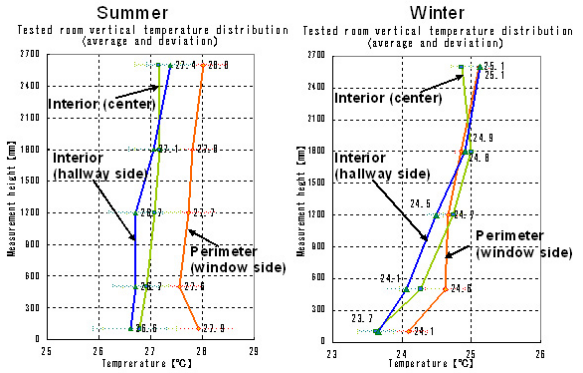
Vertical temperature distribution in representative room (summer at left, winter at right).

Comfort is different for each space-conditioned area. To examine the room temperature and temperature distribution in the experimental room (FCU, VAV detected temperature), we performed measurements (Figure 7) of temperatures at 5 different heights (100, 500, 1200, 1800, and 2600 mm) in 3 locations: on the perimeter, which is sensitive to improved envelope performance and changes in outdoor conditions, and in the interior (center and hallway side). Data on vertical temperature distribution collected from August 1, 2005 to February 5, 2006 over the space-conditioning hours of 10:00 to 20:00 are plotted in Figure 8 as averages and standard deviations of temperature variation range over the time period of the research.

In both summer and winter, the window perimeter was affected by outdoor meteorological changes. In winter, there are large temperature differences by height, making it difficult to maintain comfort.

**Figure 9:**
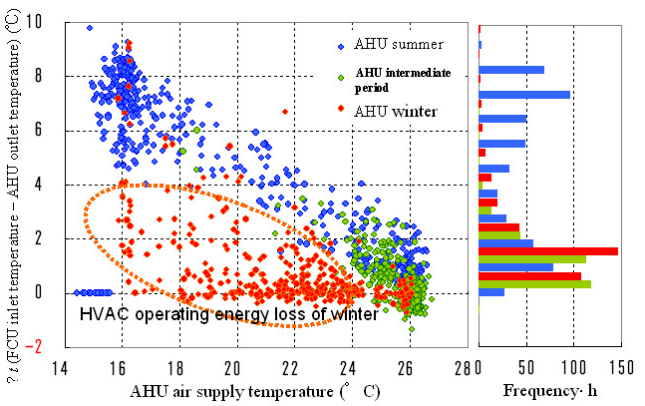
Relationship between HVAC outlets and inlets (energy loss).

We plotted the difference between FCU inlet temperature and AHU outlet temperature (Δ*t*) against the AHU air supply temperature at each measurement point (Figure 9) in order to illustrate the circumstances described above. We wanted to consider the indoor environment created by combined FCU and AHU operation, and by the design response of the AHU HVAC system including building openings. The small heat consumption at 26°C, near the upper limit of the AHU air supply temperature, suggests the effectiveness of blower operation and outside-air cooling operation in the summer and intermediate period, as well as the effectiveness of natural ventilation and of letting in cool air by opening windows and shutting down AHUs. In winter, we found an energy operating loss, caused by the effect of FCU space heating that calls for reducing AHU space cooling.

Table 1 provides the main model parameter of the tested indoor environment.

**Table 1:** Parameter of heat load computing model (CCRH when designed)

			Perimeter zone	Interior zone
Direction			Tsukuba, Ibaraki, Japan : For the true south	
Area			13.43 m^2^	199.23 m^2^
	summer	9:00	387.34 W/m^2^	140 W/m^2^; temperature 26°C, humidity 50%
		12:00	479.82 W/m^2^	140 W/m^2^; temperature 26°C, humidity 50%
		14:00	515.93 W/m^2^	140 W/m^2^; temperature 26°C, humidity 50%
		16:00	464.93 W/m^2^	140 W/m^2^; temperature 26°C, humidity 50%
	winter		1223.83 W/m^2^	134.25 W/m^2^; temperature 22°C, humidity 40%
Quantity of outdoor air intake				30.0 m^3^/(h·people)
Over hang			1500 mm	
Area of glass			61.7 m^2^	
Rate of glass area			85.1%	
Area of internal wall				Floor:189.06 m^2^, Face of wall 72.5 m^2^
Floor height			4200 mm	
Ceiling height			2700 mm	
Window shade			Minimum calorie that uses window shade 100 W/m^2^	
Heat gain from occupancy			SH69W/people, LH53W/people, Dencity of occupancy 0.17 people/m^2^	
Heat generation rate of electrical outlets			29.75 W/m^2 ^(tested room 32 W/m^2^)	
Cooling load from lighting			Illuminance 700lx, 20 W/m^2^	
Thermal capacity from			12.6 kJ/m3·°C(default)	

Table 2 gives the main measurement points, and includes detailed measurements of this indoor environment.

**Table 2:** The data of CCRH monitoring

		Tested room	Controled room	Area coverd by research
1. Outside conditions (data at 10-minute intervals)		Wall insolation (1 points), outside air temperature (2 points)		
2. Indoor environment (data at 10-minute intervals)	Tested room temperature	◦ (1 point)	◦ (5 points)	◦ (22 points)
	PMV	◦ (1 point)	×	×
	Vertical temperature distribution	◦ (5 points× 3)	×	×
	Globe thermometer	◦ (2 points)	×	×
	Window pane temperature	◦ (7 points)	×	×
	Exhaust outlet temperature	◦ (3 points)	×	×
3. State of HVAC (data at 30-minute intervals)	FCU heat and airflow amounts	◦ (1 point)	◦ (5 points)	◦ (4 points× 5;correction needed)
	AHU heat and airflow amounts	×	◦ (5 points)	◦ (4 points× 5)
	VAV operation state	◦ (Temperature and humidity 2 points, anemometers 2 points)	◦ (30 points)	◦ (6 points× 22)
4. Room electricity consumption (data at 30-minute intervals)	Lighting	◦ (By system, 3 points)	◦ (2 points)	◦ (4 points× 2)
	Electrical outlets	◦ (1 point)	◦ (1 point)	◦ (4 points)
5. Total		About 1400 points throughout building (295 points in area coverd by research)		
